# Associations Between Fetal Symptoms During Pregnancy and Neonatal Clinical Complications with Cytomegalovirus Infection

**DOI:** 10.3390/children12121690

**Published:** 2025-12-12

**Authors:** Virág Bartek, Márta Csire, Gréta Kiss, Réka Hodula, Artur Beke

**Affiliations:** 1 Department of Obstetrics and Gynecology, Semmelweis University, 1088 Budapest, Hungary; bartek.virag@stud.semmelweis.hu (V.B.); kiss.greta@stud.semmelweis.hu (G.K.); hodula.reka@stud.semmelweis.hu (R.H.); 2 Division of Virology, National Center for Epidemiology, 1097 Budapest, Hungary; csire.marta@nnk.gov.hu

**Keywords:** cytomegalovirus, pregnancy, amniocentesis, PCR, serology, ultrasound abnormalities, neonatal complications

## Abstract

**Highlights:**

**What are the main findings?**

- In cases of recent Cytomegalovirus infection, the most common ultrasound abnormality affects the gastrointestinal and nervous system.
- Based on the neonatological follow-up examinations, 15.52% newborns had complications.

**What are the implications of the main findings?**

- Accurate knowledge of the rate of ultrasound abnormalities is important for prenatal diagnosis.
- Amniotic fluid CMV PCR yields valuable information about CMV transmission, supporting decisions in newborn care.

**Abstract:**

Introduction: Primary Cytomegalovirus (CMV) infection occurs in 0.7–4.1% of all pregnancies. Our study aims to analyze the incidence rate of ultrasound anomalies, as well as CMV PCR analysis of the amniotic fluid sample obtained from amniocentesis in CMV-infected pregnancies, as well as the outcome of the pregnancies and neonatal follow-up. Methods: We analyzed cases of recent maternal CMV infections confirmed by serological testing at the Department of Obstetrics and Gynecology, Semmelweis University, between 2001 and 2023. In cases of primary CMV infection confirmed by serological testing during pregnancy, we offered amniocentesis at the genetic counseling, which was performed at the 20–21 weeks stage of the pregnancy. Results: In 130 cases of recent maternal CMV infection confirmed by serological testing, amniocentesis was performed, and a total of 11 cases (8.46%) were found to have CMV DNA in the amniotic fluid. Based on the neonatological follow-up examinations in 116 deliveries, 18 newborns had complications (15.52%); however, some cases were associated with multiple complications, resulting in a total of 33 types of complications being identified (28.45%). Among the 11 neurological complications (9.48%), we found 1 case each (0.86%) of severe inoperable intracranial space occupation, hydrocephalus, balance disorder, sleep disorder–sleep apnea, and speech development disorder. Two cases (1.72%) were found to have rigid muscles, epilepsy, and hypotonic muscles. Ophthalmological complications occurred in five cases (4.31%), such as enophthalmos, cataract, and retinopathy of prematurity (ROP), one case each, and two cases of strabism. Other complications were detected in 17 cases (14.66%). Conclusions: Because of the high incidence rate of recent CMV infection, serological testing is recommended following fetal abnormality detected by ultrasound. If a serologically confirmed new infection is diagnosed, the affected couple should be offered amniocentesis.

## 1. Introduction

Transplacental viral infections have been extensively studied in recent decades. Intrauterine fetal infections often cause spontaneous abortion or fetal abnormalities [1,2]. The various adverse effects may include the production of toxic metabolites, fetal infection, chronic endometrial infection, or chorioamnionitis. Viruses are known to be the most common pathogens that can cause fetal infection [3].

Primary cytomegalovirus (CMV) infection occurs in 0.7–4.1% of all pregnancies [4]. The most common congenital infection is caused by CMV, which affects 0.5–1% of live births in North America and Europe and 6% of live births in developing countries [5].

Despite the low sensitivity (<25%) and positive predictive value, the earliest sign of congenital CMV infection is observed during routine fetal ultrasound at 18–20 weeks of gestation. Echogenic bowel may be the first indicator of an intrauterine CMV infection [5].

The abnormalities that can be detected during an ultrasound scan of the skull can be varied. For example, ventricular or periventricular calcification, microcephaly, ventriculomegaly, cerebellar (sub)ependymal or parenchymal cysts, and cortical abnormalities, such as polymicrogyria, may occur. Migratory abnormalities may include lissencephaly, porencephaly, schizencephaly, or extensive encephalopathy, and vascular abnormalities may include lenticulostriatal vasculopathy (LSV) [2,5]. Other observable indicator ultrasound abnormalities may include amniotic fluid abnormalities, oligohydramnios or polyhydramnios, thickened placenta, calcified placenta, hepatosplenomegaly, hepatic calcification, ascites, pericardial effusion, hyperechogenic kidneys, or hydrops [4,6]. In more severe cases, fetal growth restriction (FGR), small for gestational age (SGA) may be observed [7].

In the case of a positive ultrasound result, serological testing of maternal serum samples by ELISA is recommended to determine IgM and IgG levels. The presence of IgM and IgG, combined with low IgG avidity, indicates a recent infection within 2–4 months, whereas high IgG avidity indicates an infection older than 4 months [4].

When fetal abnormalities are suspected of being infectious, and the mother has a documented primary or unknown CMV infection in the first half of pregnancy, amniocentesis is recommended by the Society for Maternal–Fetal Medicine [6]. At present, microbiological molecular testing (PCR) of amniotic fluid samples obtained by amniocentesis also allows couples to make an informed decision on whether to carry the pregnancy to term [7,8]. For the diagnosis of fetal infection, the detection of the nucleic acid of the pathogen by RT-PCR testing of amniotic fluid samples obtained during amniocentesis at 20–21 weeks of pregnancy is recommended. Sampling should be scheduled at least 6 weeks after maternal infection [1,9].

The impact of other infections on obstetric complications has been raised in the literature. Amadori et al. conducted a retrospective study comparing births in the pre-COVID period and during the COVID period (during and immediately after the lockdown). Their study found no significant differences in either prenatal or intrapartum complications [10].

Our study aims to analyze the incidence rate of ultrasound abnormalities in recent CMV infections confirmed by serological testing and to analyze the CMV-PCR results of amniotic fluid samples, as well as the outcome of pregnancies and the results of neonatological follow-up.

## 2. Materials and Methods

In our study, we analyzed cases of recent maternal CMV infections confirmed by serological testing between 2001 and 2023 at the Department of Obstetrics and Gynecology, Semmelweis University. In cases of primary CMV infection confirmed by serological testing during pregnancy, after detailed consultation with the couple, we offered amniocentesis at the genetic counseling session, which was performed during 20–21 weeks of pregnancy.

The inclusion criteria for the study were as follows: pregnant women were included if routine CMV serology during pregnancy confirmed a recent CMV infection, regardless of whether ultrasound (US) abnormalities were present at the time or appeared later. Additionally, cases were included if CMV serology was conducted due to suspicious findings on an ultrasound, and the serology results confirmed a recent CMV infection. Furthermore, all cases were eligible for inclusion if an amniocentesis was performed for CMV PCR examination. These criteria ensured that the study focused on pregnancies with confirmed CMV infections and relevant diagnostic procedures.

On the other hand, the exclusion criteria were clearly defined. Pregnant women were excluded from the study if they did not request amniotic fluid sampling, which is a key diagnostic procedure for CMV PCR testing. Moreover, women who did not provide consent to participate in the study were also excluded. This ensured that the study only included participants who actively agreed to undergo the necessary procedures and contribute to the research. By setting these criteria, the study aimed to maintain a focused and ethical approach to investigating CMV infections during pregnancy.

### 2.1. Ultrasound Scans

The ultrasound examinations were performed in the Ultrasound Laboratory of the Department of Obstetrics and Gynecology using a Medison Sonoace X8 (Medison Co., Ltd., Seoul, Republic of Korea), Samsung Medison UGEO H60 (Samsung Medison Co., Ltd., Seoul, Republic of Korea), Samsung Medison WS80A (Samsung Medison Co., Ltd., Seoul, Republic of Korea), and Philips^®^ HD 11XE (Philips Ultrasound, Bothell, WA, USA) ultrasound devices. The examinations were performed in accordance with the professional protocols developed by the Hungarian Obstetric-Gynecological Ultrasound Society (Obstetric pregnancy transabdominal ultrasound examination-10 February 2003; Fetal echocardiography-10 February 2003; Ultrasound examinations recommended during pregnancy-10 February 2003) and the current Health Professional Guidelines (On diagnostic and basic ultrasound screening examinations in early pregnancy-2016).

The significant fetal anomalies include fetal cranial abnormalities (ventriculomegaly, cerebral calcification, ventricular dilatation III and IV, and microcephaly), subcutaneous edemas (NT, hydrops, anasarca, hygroma), cardiac and thoracic abnormalities (pericardial effusion, pleural effusion, narrow thorax), abdominal anomalies (echogenic bowel, dilated bowel, echogenic liver, perihepatic fluid, ascites, pyelectasia), and placental anomalies (cystic placenta, thickened placenta, premature calcification, amniotic bands). Genetic counseling was performed in cases of ultrasound signs suspicious of infection, during which the expectant mother/couple was informed in detail about the possible risks and complications of the infection. Serological testing of maternal serum samples by ELISA was performed to determine the specific IgM and IgG levels of CMV, and in the case of recent infection, the CMV IgG avidity value. If the serological result was positive or inconclusive, or if a new or worsening lesion was seen in the control ultrasound examination, amniocentesis was recommended to detect possible intrauterine transmission.

### 2.2. CMV PCR Examinations

The collected amniotic fluid (AF) samples were transported to the Genetics Laboratory, where molecular microbiological testing (PCR) of AF samples for processing was performed for the detection of CMV DNA. The samples were stored at −20 °C until processing. DNA isolation from amniotic fluid was carried out by phenol/chloroform extraction or QIAamp DNA Mini Kit (QIAGEN, Vienna, Austria).

We performed nested PCR with primers specific for the DNA polymerase genes of lymphotropic herpesviruses [11]. We performed nested PCR with specific oligonucleotide primers designed for the conserved region of the DNA polymerase gene. Based on international results, the sensitivity of the nested PCR assay was estimated to be between 200 and 300 copies/mL. A multiplex PCR designed for the amplification of DNA of HSV-1 and 2, CMV, and VZV [12] was performed to isolate herpesviruses. CMV DNA-positive controls were prepared from human herpesvirus 5 strain AD-169 (ATCC: VR-538) grown on primary human fibroblast cells.

An Artus CMV LC PCR Kit (QIAGEN, catalog number 4503063, Vienna, Austria) was used for quantitative CMV PCR. The quantification of viral DNA in the amniotic fluid is the most appropriate diagnostic method for fetal HCMV infection. AF qPCR has a sensitivity of approximately 80–90% and a specificity of 100% when sampled at least 8 weeks after the onset of maternal infection and at 20–21 weeks of gestation [13].

### 2.3. Neonatal Follow-Up

After birth, a neonatological, microbiological, pediatric neurological and ophthalmological, and audiological examination was carried out. The neonatal follow-up was carried out in the Neonatal Follow-up Unit of our clinic, where, in addition to general tests, regular neurological, ophthalmological, and audiological examinations were performed.

## 3. Results

Between 2001 and 2023, 130 samples of amniotic fluid were subjected to molecular microbiological testing (PCR) for human CMV DNA at the Department of Obstetrics and Gynecology, Semmelweis University, Budapest. The mean maternal age of the cases was 31.62 ± 5 years. Of the 123 pregnancies (94.61%) that we know of that resulted in a birth, 116 infants were born. Based on the processing of birth data, we determined the proportion of preterm and term newborns. The proportion of premature newborns was found to be 7.76% (9 cases), while the other 107 cases (92.24%) were mature newborns.

### 3.1. Processing Our Ultrasound Results

The first table contains the indications for amniotic fluid sampling and CMV PCR tests (Table 1). In 67 of the 130 cases, routine CMV serological screening confirmed primary CMV infection; in 51 of these cases, no ultrasound abnormality was observed during pregnancy, and in 16 cases ultrasound abnormality developed later. In 63 of the 130 cases, CMV serology was performed due to a suspicious ultrasound abnormality: in 27 cases, one; in 27 cases, two ultrasound abnormalities were detected in the fetus; in 6 cases, three; in 2 cases, four; and in 1 case, five ultrasound abnormalities were detected.

First, the prevalence and percentage distribution of fetal abnormalities observed by ultrasound are presented.

**Figure 1:**
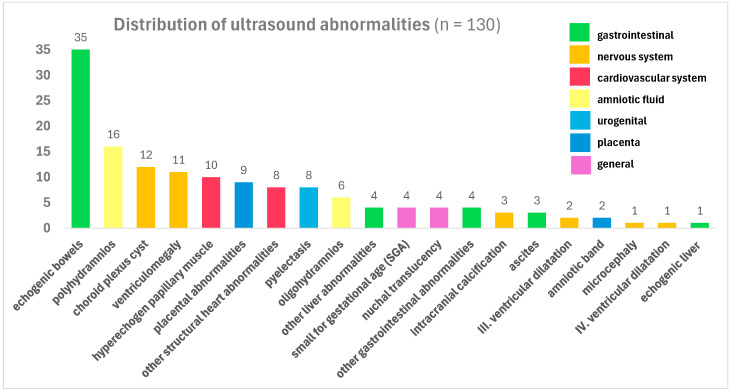
Distribution of ultrasound abnormalities in descending order, with different colors for each organ system.

The ratio of ultrasound deviations was analyzed based on the total number of cases (130) and grouped by organ system. Figure 1 shows the distribution of ultrasound abnormalities.

In the literature, the most common ultrasound abnormalities are echogenic bowel, issues with craniospinal development (calcifications or enlarged ventricles), microcephaly, growth restriction, poly- or oligohydramnion, problems with the placenta, hepatosplenomegaly with or without intrahepatic calcification, ascites, and hydrops [4].

The presence of abnormal amniotic fluid during ultrasound examination can be a cause for concern. In Table 2, it is observed that of the 130 cases accumulated over the years, 17 cases (13.08%) presented polyhydramnios, while oligohydramnios occurred in only 6 cases (4.62%). Intrauterine growth restriction was also detected in only a small number of cases (4 cases, 3.08%).

Table 3 summarizes fetal anatomical differences.

In the ultrasound diagnosis of cytomegalovirus infection, cranial abnormalities are considered warning signs. Out of 130 cases, cumulatively 38 cases (29.23%) showed cranial abnormalities, most commonly cerebral ventriculomegaly was the dominant lesion, detected in 14 cases (10.77%). In six cases, the lesion was bilateral. Interestingly, the choroid plexus cyst, classified as a minor anomaly, was the second most common abnormality in 13 cases (10%), of which 3 cases were bilateral. Cerebral ventricular dilatation III was found in two cases (1.54%), but microcephaly and cerebral ventricular dilatation IV were found in only one case each (0.77%). Cerebral calcification was detected in two cases (1.54%). Posterior fossa cyst and cysterna magna dilatation were not detected in any of the cases.

The next part of the table illustrates the heart and chest abnormalities. Of the 130 cases, 15 (11.53%) were cardiac abnormalities, while chest and lung abnormalities were found in a smaller proportion of cases, in only 3 cases (2.3%). Of the significant number of cardiac abnormalities, hyperechogenic papillary muscle was dominant, found in 10 cases (7.69%).

Dilated cardiac cavity and other structural abnormalities were diagnosed in four cases (3.08%), but pericardial effusion was found in only one fetus (0.77%). Among the thoracic abnormalities, a narrow chest was seen in two cases (1.54%), while pleural effusion was seen in only one case (0.77%).

A further part of Table 3 summarizes and represents our results on abdominal anomalies. Out of 130 cases, 58 cases (44.62%) presented alarming abdominal abnormalities. Renal involvement was relatively frequent, with pyelectasia confirmed in eight cases (6.15%). Liver abnormalities were detected less frequently in only seven cases (5.38%). Of these, inhomogeneous liver structure was found in two fetuses (1.53%). Echodense liver was found in three cases (2.31%). Hepatomegaly and perihepatic fluid were diagnosed in one case each (0.77%).

Particular attention should be paid to lesions involving the intestines, which were present in 39 cases (30%). In 26.92% of our total patient population, 35 cases of echogenic bowel were detected, making it the most prevalent abnormality in our table. Ascites was detected in four cases (3.07%), dilated bowels were detected in three cases (2.31%), while no visible gastric filling was detected in one case (0.77%).

The fetal subcutaneous edema occurs in a smaller proportion of cases. Edema of the nuchal fold was described in 4 cases (3.08%). In contrast, hydrops, anasarca, and hygroma did not occur in any of the fetuses.

Finally, the anomalies of the placenta are shown in Table 4. Abnormalities in the placenta exposed to transplacentally transmitted pathogens may indicate possible infection. In our studied cases, we encountered nine cases (6,92%) of placental abnormalities, most commonly cystic parts of the placenta, detected in seven cases (5.38%).

Placental calcification and thickened placenta were confirmed in one case each (0.77%). Amniotic bands were detected in two cases (1.54%).

Less characteristic fetal circulation anomalies were found in four cases (3.07%). A reverse A wave was detected in the ductus venosus, and singular umbilical artery (SUA) was described in two cases each (1.53%).

### 3.2. Processing Our PCR Results

**Figure 2:**
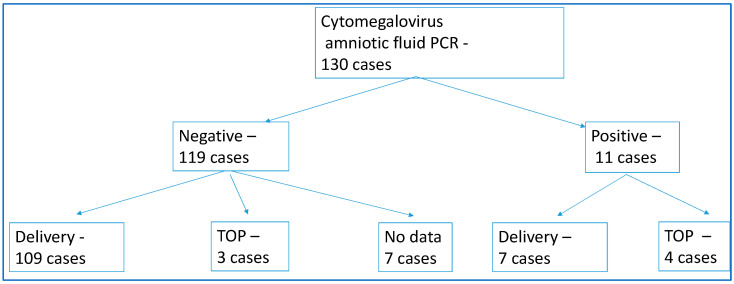
The outcomes of the pregnancies. TOP—termination of pregnancy.

Amniocentesis was performed in 130 cases of suspected recent maternal CMV infection. The results of the evaluated PCR findings and the outcome of the pregnancies were carefully analyzed. The results are shown in Table 5, which demonstrates the number of cases and their percentage. Of the 130 serologically confirmed cases of recent CMV infection, a total of 11 cases (8.46%) were found to have CMV DNA in amniotic fluid (Figure 2). After discussing the results of the tests performed and the risks involved, the affected mother or couple made an informed decision about the fate of the pregnancy. Of the 11 cases where PCR of amniotic fluid was positive, 7 (63.63%) couples chose to give birth, while 4 (36.36%) chose to terminate the pregnancy. Where the amniocentesis PCR result was negative, a significantly higher proportion of couples, 109 cases (91.59%) out of 119 pregnancies, decided to carry the pregnancy to term. TOP (termination of pregnancy) was performed in only three cases (2.52%). The outcome of seven pregnancies is currently unknown, while spontaneous miscarriage did not occur in our study.

This test is also important because a couple with a negative result is much more likely to decide to carry the pregnancy to term.

### 3.3. Neonatal Follow-Up

**Figure 3:**
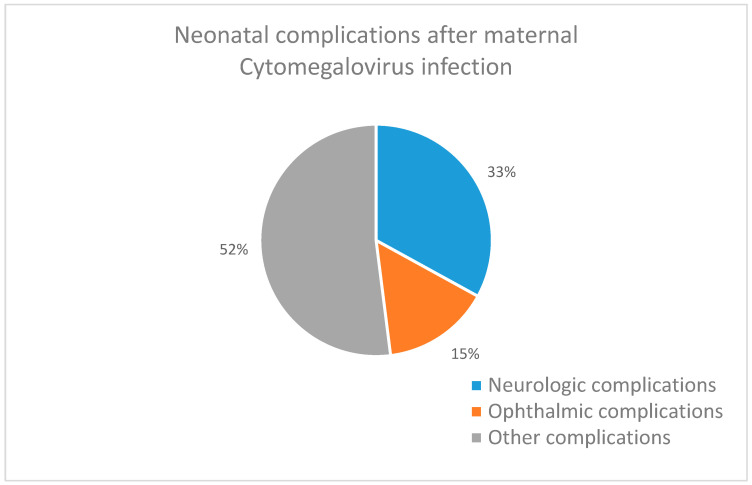
Neonatal complication after maternal CMV infection.

When examining the outcome of pregnancies, we processed the incidence of neurological, ophthalmological, and other complications in newborns. The rates of complications are shown in Figure 3. The cases are illustrated in Table 6.

### 3.4. Association Between Ultrasound Abnormalities and Neonatal Complications

Fisher’s exact test was used to examine whether there was a difference between a positive ultrasound finding and later complications. A significant result was found between polyhydramnios and later complications (0.003) (Table 7).

Based on the neonatological follow-up examinations in 116 deliveries, 18 newborns had complications (15.52%), and 33 types of complications were identified (28.45%).

Among the 11 neurological complications (9.48%), we found 1 case each (0.86%) of severe inoperable intracranial space occupation, hydrocephalus, balance disorder, sleep disorder–sleep apnea, and speech development disorder. Two cases (1.72%) were found to have rigid muscles, epilepsy, and hypotonic muscles. Ophthalmological complications occurred in five cases (4.31%): enophthalmos, cataract, and retinopathy of prematurity (ROP) in one case each, and two cases of strabismus. Other complications were detected in 17 cases (14.66%).

There was one case (0.86%) each of scoliosis, autoimmune disease, cardiomegaly, insufficient weight gain, cumulative food allergy, pneumonia, and chronic peritonitis. A higher proportion of 2 cases (1.72%) had icterus, multiple malformations, hyperinsulinemic hypoglycaemia, and pulmonary edema. Unfortunately, severe neonatal conditions resulted in postpartum exitus in two cases (1.72%).

## 4. Discussion

Based on data from the literature, we obtained results similar to previous research. However, there were differences in our study, which we will highlight below.

### 4.1. Congenital Infection

In a 2017 study by Simonazzi et al., 239 cases of pregnant women were reviewed, of which, in all 239 cases, amniocentesis was performed, with positive results in 13.4% (32 cases) and negative results in 86.6% (207 cases) [7]. In our own study of 130 cases, congenital infection was diagnosed with CMV PCR in 11 cases, corresponding to 8.46%.

### 4.2. Ultrasound Abnormalities

When comparing the ultrasound abnormalities detected in recent CMV infections with the literature, the incidence rate of ultrasound abnormalities in confirmed congenital CMV infections, based on the 2016 publication by Hughes and Gyamfi-Bannerman (Society for Maternal–Fetal Medicine), was as follows: microcephaly in 14.5% of cases, echogenic bowel in 4.5–13%, IUGR in 1.9–13%, ventriculomegaly in 4.5–11.6%, ascites in 8.7%. Other abnormalities such as cerebral calcification, subependymal cysts, pericardial effusion, hyperechogenic kidney, hepatomegaly, enlarged or calcified placenta, hepatic calcification, and hydrops are also reported in the publication [6]. In our own studies, we detected microcephaly, ventriculomegaly, and IUGR in 9.09% of the 11 confirmed with CMV PCR congenital CMV infected cases, echogenic bowel in 45.45% and ascites in 18.18%. It can be seen that the percentage distribution of the former three abnormalities is in accordance with what has been reported in the publication, whereas the latter two abnormalities, echogenic bowel and ascites, occurred in a significantly higher proportion in our study.

Simonazzi et al. described 29 cases of 2nd trimester ultrasound abnormalities out of 588 newborns, of which 8 were cranial and 21 extracranial abnormalities. The eight cranial abnormalities included the following cases: four ventriculomegaly (13.79%), two hyperechogenic halo, one corpus callosum hypoplasia, and one choroid plexus cyst (3.45%). Extracranial abnormalities included the following cases: nine echogenic bowel (31.03%), nine renal malformations (31.03%), two cleft lip and palate, and one abdominal cyst [7]. Compared to our data, we reached different results. Compared to the published data, ventriculomegaly was observed in a slightly lower proportion (10.77%) of 130 cases of fresh CMV infection detected by serological testing, whereas choroid plexus cysts occurred in a higher proportion of 10%. Echogenic bowel was detected in a nearly similar percentage of 26.92%, but renal abnormalities in a significantly lower proportion of only 6.15%. The average maternal age of 31 ± 5 years was also determined by the researchers. This is in complete agreement with the data of our study, which was 31.62 ± 5 years.

It was interesting to observe that the plexus choriodeus cyst and the hyperechoic papillary muscle, described as a minor sign in prenatal diagnostics, occurred more often in our results than the ratio observed in Hungary. In Hungary, choroid plexus cysts can be observed in 4.1% of pregnancies during the second-trimester ultrasound examination; in the present study, the occurrence rate of choroid plexus cysts in the case of recent maternal CMV infection was 10%.

A similar observation was made in the case of the hyperechoic papillary muscle. Its prevalence in the Hungarian population is 3.5%. On the other hand, based on our observations, the incidence of hyperechoic papillary muscle in the case of recent maternal CMV infection was 7.69%.

The two minor anomalies did not constitute an indication for CMV serology testing; however, their more frequent occurrence raises the need for the investigation of additional causes in the background of the minor deviations, which are currently described as still of unknown origin in most cases.

### 4.3. Pregnancy Outcome

Picone et al. studied 238 primary CMV-infected pregnant women; amniocentesis was performed in 36.1% of cases. In 18 cases (7.56%), abortion was performed due to an ultrasound abnormality [8]. This value exceeds our obtained values, with TOP in 7 of our 130 cases, representing 5.38%. Furthermore, it was mentioned that 5.5% of the asymptomatic cases developed hearing loss; however, based on our own data, we did not have any hearing complications. This may be due to the small number of subjects we worked with or due to the nature of the hospital center; patients were often lost to follow-up. We suggest that there were affected patients due to the frequency of hearing loss, but we could not confirm this.

In a 2017 study by Simonazzi et al., 239 cases of pregnant women were reviewed, of which 222 (92.9%) resulted in delivery and 17 (7.1%) in 2nd trimester termination [7]. According to our own study data, pregnancy outcomes were as follows: 116 cases (89.23%) ended in delivery, 7 cases (5.38%) ended in TOP, and 7 cases had unknown outcomes. We can conclude that our results are similar to those reported in the publication.

### 4.4. Small for Gestational Age (SGA)

A 2014 study by Simonazzi et al. looked at the rate of newborns with SGA and long-term complications. Of the initial 848 primary CMV-infected pregnant women, 157 were excluded, and 103 patients were lost to follow-up. Finally, 588 live newborns remained in the study. A total of 35 cases of SGA were found (6%), and cCMV infection was diagnosed in 119 cases, corresponding to 20%. SGA was found in 6.7% of infected cases and 5.7% of non-infected cases [14]. In contrast, in our study, cCMV infection was detected in 11 cases, 8.46%, which is far below 20%. We found a total of four cases of SGA, which represents 3.08%, a result that also falls under the 6% reported by the researchers overall. We diagnosed SGA in 3 of our 119 non-infected cases (2.52%) and in 1 of our 11 infected cases, which is 9.09%. The former result is below the data reported in the literature, while the latter is well above it.

### 4.5. Premature Birth Rate

We compared our results on preterm births with data published by the Hungarian Statistical Office (KSH). According to the publication, since 1991, the rate of preterm births has decreased gradually to 8.3%. Among the patients we studied, the rate of preterm births was 10 cases, or 8.62%, which corresponds with the national average and is in line with the literature reported by Davis et al. [4]. According to the researchers, nearly one-quarter to one-third of symptomatic congenital CMV-infected infants are preterm, which may explain the higher result we found [7,15].

### 4.6. Neonatal Follow-Up

Barton et al. estimated that the prevalence of long-term neurological complications ranges from 5 to 15% in children with asymptomatic congenital CMV infection, while the prevalence in symptomatic cCMV-infected children can be as high as 36–90% [5]. In our study, there were 11 neurological complications (9.48%), 1 of which resulted in death within 2 h of birth.

Capretti et al. report on neonatal and long-term ophthalmological complications in symptomatic and asymptomatic cCMV-infected patients. They studied 48 cases, of which 18 were symptomatic. In 78% (14 cases) of symptomatic cases, neurological imaging abnormalities were found, and cerebral calcification and ventriculomegaly were identified in 3 (21.43%) cases. In the remaining cases, white matter abnormalities and pseudocysts were observed [16]. In our study, white matter abnormalities and pseudocysts were not observed. Of the 11 cases we confirmed, ventriculomegaly was detected in 1 case (9.09%), which is far below the publication data, and no brain calcification was found.

Jin et al. studied long-term ophthalmological complications in 237 congenital CMV-infected patients [17]. Of the 77 symptomatic patients, severe visual loss occurred in 18.2% (14 cases), strabismus in 23.4%, chorioretinal scarring in 19.5%, cortical visual loss in 14.3%, nystagmus in 14.3%, and optic nerve atrophy in 11.7% [16]. In our own research, strabismus occurred in two cases, and enophthalmos, ROP, and cataracts in one case each.

Picone et al. reported that 5.5% of asymptomatic cases developed hearing loss, but compared to their data, no hearing complications occurred in our study [8].

## 5. Conclusions

In summary, our study shows that ultrasound abnormalities in recent CMV infection can be diverse. Polyhydramnios was a notable abnormality. Choroid plexus cysts and ventriculomegaly were the most common cranial abnormalities, hyperechogenic papillary muscle was the most common cardiac abnormality, echogenic bowel and pyelectasis were the most common abdominal abnormalities, and cystic parts indicating placental involvement were the most common.

Our investigations have led us to the following conclusions. Because of the high prevalence of recent CMV infection, serological testing is recommended following a fetal abnormality detected by ultrasound. If the mother is considered susceptible to the infection, the genetic counseling should also include prevention options. If a serologically confirmed new infection is diagnosed, the couple concerned should be offered amniocentesis to detect possible intrauterine transmission. Information on fetal involvement, obtained by molecular microbiological testing of amniotic fluid samples, has a major impact on the decision of the affected mother to have the pregnancy delivered. With a negative result, a much higher proportion of couples decided to carry the pregnancy to term, and the number of induced abortions was significantly reduced. Overall, depending on the results of the tests, genetic counseling and further testing may be worthwhile. In the past, there was a higher rate of terminations following the detection of ultrasound abnormalities of infectious origin, which has now fallen as a result of more accurate DNA-based PCR tests. Diagnosing infected babies as soon as possible allows for early treatment and monitoring. Close follow-up of symptomatic and asymptomatic newborns and continuous screening tests can help to detect early the various long-term complications. Severely infected children with central nervous system involvement will require the appropriate multidisciplinary support.

## Figures and Tables

**Table 1:** Indications for amniotic fluid CMV PCR tests (130 cases).

	Recent CMV Cases (*n*)			US Later Became More Severe	The Number of Ultrasound Abnormalities Increased
Routine CMV serology screening	67				
-Without US abnormalities		51			
-With later US abnormality		16			
1 US abnormality			10		
2 US abnormalities			5		
3 US abnormalities			1		
4 US abnormalities			0		
CMV serology because of suspicious ultrasound	63				
1 US abnormality			27	7	2
2 US abnormalities			27	8	3
3 US abnormalities			6	1	1
4 US abnormalities			2	1	0
5 US abnormalities			1	1	0
Total	130			18	6

Abbreviation: US—ultrasound.

**Table 2:** Amniotic fluid abnormalities and fetal growth restriction (FGR) detected in cases of recent maternal Cytomegalovirus infection at the time of amniocentesis and cumulatively throughout the pregnancy (130 cases).

	At the Time of GAC		Cumulatively Throughout the Pregnancy		
	Cases (*n*)	%	Cases (*n*)	%	*p*
Polyhydramnios	9	6.92%	17	13.08%	NS
Fetal growth restriction (FGR)	3	2.31%	4	3.08%	NS
Oligohydramnios	1	0.77%	6	4.62%	*p* < 0.05

Abbreviations: GAC—genetic amniocentesis, NS—not significant.

**Table 3:** Fetal anomalies detected in cases of recent maternal Cytomegalovirus infection at the time of amniocentesis and cumulatively throughout the pregnancy (130 cases).

	At the Time of GAC		Cumulatively Throughout the Pregnancy		
	Cases (*n*)	%	Cases (*n*)	%	*p*
**Cranial anomalies**					
Choroid plexus cyst	13	10.00%	13	10.00%	
Ventriculomegaly	8	6.15%	14	10.77%	NS
Intracranial calcification	2	1.54%	2	1.54%	
III. ventricular dilatation	1	0.77%	2	1.54%	NS
IV. ventricular dilatation	1	0.77%	1	0.77%	
Microcephaly	1	0.77%	1	0.77%	
Other cranial anomalies	4	3.08%	5	3.85%	NS
Total	30	23.08%	38	29.23%	NS
**Cardiac and thoracic anomalies**					
Hyperechoic papillary muscle	9	6.92%	10	7.69%	NS
Structural deviation, enlarged cavities	6	4.62%	4	3.08%	NS
Narrow chest	2	1.54	2	1.54%	
Chest fluid	1	0.77%	1	0.77%	
Pericardial fluid	0	0.00%	1	0.77%	NS
Total	18	13.85%	18	13.85%	
**Abdominal anomalies**					
***Intestinal***					
Echogenic bowels	33	25.38%	35	26.92%	NS
Gastric filling is not visible	1	0.77%	1	0.77%	
Dilated bowels	0	0.00%	3	2.31%	NS
***Liver***					
Echogenic liver	2	1.54%	3	2.31%	NS
Perihepatic fluid	1	0.77%	1	0.77%	
Inhomogeneous structure, hyperdense reflections	1	0.77%	2	1.54%	NS
Hepatomegaly	0	0.00%	1	0.77%	NS
***Ascites***	3	2.31%	4	3.08%	NS
***Kidney anomalies***					
Pyelectasia	4	3.08%	8	6.15%	NS
Total	45	34.62%	58	44.62%	NS
**Subcutaneous edema**					
NT (nuchal translucency)	4	3.08%	4	3.08%	
Hydrops	0	0.00%	0	0.00%	
Anasarca, edema	0	0.00%	0	0.00%	
Hygroma	0	0.00%	0	0.00%	
Total	4	3.08%	4	3.08%	

NS—not significant.

**Table 4:** Placental anomalies in case of recent Cytomegalovirus infection at the time of amniocentesis and cumulatively throughout the pregnancy (130 cases).

	At the Time of GAC		Cumulatively Throughout the Pregnancy		
	Cases (*n*)	%	Cases (*n*)	%	*p*
Cystic placenta	6	4.62%	7	5.38%	NS
Thickened placenta	0	0.00%	1	0.77%	NS
Calcified placenta	0	0.00%	1	0.77%	NS
Total	6	4.62%	9	6.92%	NS

NS—not significant.

**Table 5:** The couple’s decision and the result of amniotic fluid sampling in case of recent CMV infection (*n* = 130).

	Amniotic Fluid PCR Positive		Amniotic Fluid PCR Neg		Total	
	Cases (*n*)	%	Cases (*n*)	%		
Delivery	7	63.64%	109	97.32%	116	94.31%
Termination of pregnancy	4	36.36%	3	2.68%	7	5.69%
Spontaneous miscarriage	0	0.00%	0	0.00%	0	0.00%
*The outcome of pregnancy is known-Total*	11		112		123	
*The outcome of the pregnancy is unknown*	0		7		7	
Total	11		119		130	

**Table 6:** Neonatal complications after maternal CMV infection during pregnancy (116 deliveries).

	Cases (*n*)	%
**Neonatal neurologic complications**		
Rigid muscles	2	1.72%
Epilepsy	2	1.72%
Hypotonia (muscle weakness)	2	1.72%
Developmental speech disorder (did not speak until the age of 4) *	1	0.86%
Severe, inoperable intracranial process	1	0.86%
Hydrocephalus	1	0.86%
Balance disorder	1	0.86%
Sleep disorder, sleep apnea	1	0.86%
Total	11	9.48%
**Neonatal ophthalmic complications**		
Strabismus	2	1.72%
Enophthalmus	1	0.86%
Retinopathia prematurorum (ROP)	1	0.86%
Cataracts	1	0.86%
Total	5	4.31%
**Other complications**		
Icterus	2	1.72%
Multiple malformation	2	1.72%
Hyperinsulinemic hypoglycemia	2	1.72%
Postpartum exit	2	1.72%
Pulmonary edema	2	1.72%
Scoliosis	1	0.86%
Autoimmune disease (at age 2)	1	0.86%
Cardiomegaly	1	0.86%
Insufficient weight gain	1	0.86%
Cumulative food allergy	1	0.86%
Pneumonia (postpartum)	1	0.86%
Chronic peritonitis	1	0.86%
Total	17	14.66%

* Patient had hearing impairment.

**Table 7:** Association between neonatal complications and fetal ultrasound malformations.

	Fetal Malformations on Ultrasound (116 Livebirths)					
	Negative		Positive		Total	Fisher’s Exact Test
	Cases (*n*)	%	Cases (*n*)	%		
	Subcutaneous edema					
negative	95	97.94%	2	2.06%	97	
positive	19	100.00%	0	0.00%	19	
Total	114	98.28%	2	1.72%	116	1.000
	Craniospinal malformations					
negative	77	79.38%	20	20.62%	97	
positive	14	73.68%	5	26.32%	19	
Total	91	78.45%	25	21.55%	116	0.554
	Cardiovascular malformations					
negative	86	88.66%	11	11.34%	97	
positive	15	78.95%	4	21.05%	19	
Total	101	87.07%	15	12.93%	116	0.267
	Pulmonary malformations					
negative	97	100.00%	0	0.00%	97	
positive	18	94.74%	1	5.26%	19	
Total	115	99.14%	1	0.86%	116	0.164
	Urogenital malformations					
negative	87	89.69%	10	10.31%	97	
positive	18	94.74%	1	5.26%	19	
Total	105	90.52%	11	9.48%	116	0.689
	Hepatic malformations					
negative	94	96.91%	3	3.09%	97	
positive	19	100.00%	0	0.00%	19	
Total	113	97.41%	3	2.59%	116	1.000
	Gastrointestinal malformations					
negative	71	73.20%	26	26.80%	97	
positive	11	57.89%	8	42.11%	19	
Total	82	70.69%	34	29.31%	116	0.269
	Placenta abnormalities					
negative	92	94.85%	5	5.15%	97	
positive	16	84.21%	3	15.79%	19	
Total	108	93.10%	8	6.90%	116	0.122
	Polyhydramnios					
negative	89	91.75%	8	8.25%	97	
positive	12	63.16%	7	36.84%	19	
Total	101	87.07%	15	12.93%	116	0.003
	Oligohydramnios					
negative	93	95.88%	4	4.12%	97	
positive	17	89.47%	2	10.53%	19	
Total	110	94.83%	6	5.17%	116	0.254
	SGA					
negative	96	98.97%	1	1.03%	97	
positive	18	94.74%	1	5.26%	19	
Total	114	98.28%	2	1.72%	116	0.302

## Data Availability

The original contributions presented in this study are included in the article. Further inquiries can be directed to the corresponding author.

## Abbreviations

The following abbreviations are used in this manuscript:

CMV	cytomegalovirus
cCMV	congenital cytomegalovirus
ROP	retinopathy of prematurity
LSV	lenticulostriatal vasculopathy
PCR	polymerase chain reaction
SGA	small for gestation age
